# Practical recommendations for fertility preservation in women by the *Ferti*PROTEKT network. Part II: fertility preservation techniques

**DOI:** 10.1007/s00404-017-4595-2

**Published:** 2017-11-27

**Authors:** Michael von Wolff, A. Germeyer, J. Liebenthron, M. Korell, F. Nawroth

**Affiliations:** 1 Division of Gynaecological Endocrinology and Reproductive Medicine, Medical University of Berne, Berne, Switzerland; 2 Department of Gynaecological Endocrinology and Fertility Disorders, Medical University of Heidelberg, Heidelberg, Germany; 3 Department of Gynaecological Endocrinology and Reproductive Medicine, Medical University of Bonn, Bonn, Germany; 4 Department of Obstetrics and Gynaecology, Johanna Etienne Hospital of Neuss, Neuss, Germany; 5 Centre for Infertility, Prenatal Medicine, Endocrinology and Osteology, Amedes Hamburg, Hamburg, Germany; 6 Division of Gynaecological Endocrinology and Reproductive Medicine, University Women’s hospital, Inselspital Bern, Effingerstrasse 102, 3010 Bern, Switzerland

**Keywords:** Fertility preservation, Ovarian tissue, Oocyte ovarian stimulation, GnRH agonists

## Abstract

**Purpose:**

In addition to guidelines focusing on scientific evidence, practical recommendations on fertility preservation are also needed.

**Methods:**

A selective literature search was performed based on the clinical and scientific experience of the authors. This article (Part II) focuses on fertility preservation techniques. Part I, also published in this journal, provides information on disease prognosis, disease-specific therapy, and risks for loss of fertility.

**Results:**

Ovarian stimulation including double stimulation and freezing of oocytes is the best-established therapy providing live birth chances in women < 35 years with high ovarian reserve of around 30–40%. Ovarian tissue freezing is especially useful in young women with good ovarian, if spontaneous conception is favoured and if < 1 week until chemotherapy is provided. Data on success rates are still limited, but this further evolving technique will possibly reach similar success rates as ovarian stimulation. GnRH agonists seem to reduce the risk of premature ovarian failure up to 50%; however, the effect is possibly not long-lasting. Ovarian transposition can easily be combined with freezing of ovarian tissue and is the preferred technique before pelvic radiotherapy. Other techniques, such as in vitro maturation, are limited to women with high ovarian reserve and remain less effective. In addition, procedures such as in vitro growth of follicles, etc. are still experimental.

**Conclusions:**

Fertility preservation in women provides realistic chances of becoming pregnant. The choice of technique needs to be based on the time required, the woman’s age, its risks and efficacy, and the individual preference of the patient.

## Introduction

Increasing survival rates in patients affected by oncological disease and advances in reproductive medicine have led to the development and increasing use of various fertility preservation techniques. Meanwhile, improving data and optimization of the available techniques have allowed a realistic portrayal of the efficacy and risks of the most commonly used methods, as well as recommendations for the use of the techniques, alone or in combination. Several guidelines and recommendations have been published in Europe [1], the United States [2], and elsewhere. These guidelines mainly focus on scientific evidence, but are less practically orientated.

We have, therefore, prepared an update of practical recommendations published in 2011 by the *Ferti*PROTEKT network, a network and society of physicians and biologists specializing in fertility preservation in Germany, Austria, and parts of Switzerland [3].

As the topic has become too broad for one single paper, we have prepared two articles. Part I focuses on the diseases and provides information essential in decision making for or against fertility preservation, such as prognosis of the disease and disease-specific therapy and risks for loss of fertility. In this second article, Part II, we provide recommendations specifically on the fertility preservation techniques.

## Ovarian stimulation and freezing of oocytes

### Background

The decisive factor in ovarian stimulation is maximization of the oocyte yield and minimization of the complication rate, so that oncological treatment can be started immediately after follicular aspiration. It should be noted that fertilized oocytes can only be transferred to the woman after the consent of both partners, which is why preserving some oocytes in an unfertilized state should be considered, even in the case of a stable partnership. Ovarian stimulation can now be initiated at any time during the menstrual cycle [4–7]. In addition, double stimulation [8] and stimulation directly after ovarian tissue removal are also possible [9].

### Efficacy

The number of oocytes collected depends on the age of the patient and the underlying ovarian reserve, which varies individually. According to the *Ferti*PROTEKT registry, the mean number of oocytes collected in 809 women according to age was ≤ 30 years 12.9, 31–35 years 12.3, 36–40 years 9.0, and 5.7 in women aged > 40 years [10].

Using the number of oocytes which were collected prior to chemotherapy and successfully fertilized, a *Ferti*PROTEKT study [11] calculated the theoretical birth rate depending on the age of the woman using 125 follicular aspirations (Table 1). First case series confirm these calculated success rates. In 90 women who had cryopreserved oocytes, 196 embryo transfers were performed, which led to the birth of 35 children (birth rate 38.9% per patient) [12]. Since the number of frozen oocytes is decisive for the later chances of conception, double stimulation could increase the number of cells.

**Table 1:** Estimated live birth rate after ovarian stimulation, based on the number of retrieved oocytes and registry data ([11], modified)

Age of the women at the time of ovarian stimulation	Median number of collected oocytes	Median number of fertilized oocytes	Estimated live birth rate (%)
< 26	13.5	8.6	40
26–30	11.3	7.3	35
31–35	11.0	6.1	30
36–40	8.3	5.1	25

Cryopreservation of oocytes is now usually performed by vitrification. Rienzi et al. [13] compared oocytes that were directly fertilized after removal and freshly transferred or those that were previously cryopreserved in an unfertilized state. Pregnancy rates were not significantly different. These data were confirmed in a later meta-analysis [14]. The effectiveness of oocyte vitrification has also been confirmed in cancer patients [15]. In 11 women whose oocytes were removed before gonadotoxic therapy at an average age of 35.6 years (30–41 years), thawed, and fertilized, the oocyte survival rate was 92%, the fertilization rate 77%, and the implantation rate 32%. A pregnancy occurred in 7 of the 11 women and a live birth resulted in 4 (36%).

### Risks

Ovarian stimulation can lead to side effects caused by the medication, as well as complications during the puncture. However, clinically relevant bleeding during follicular aspiration or inflammation is rare. High-grade overstimulation amongst *Ferti*PROTEKT network patients occurred only once in 708 stimulations [6].

### Practical approach

The standard protocol for stimulation is the antagonist protocol with ovulation induction using GnRH agonists (triptorelin 0.2 mg s.c.) to minimize the risk of ovarian hyperstimulation syndrome (OHSS) [16].

#### Random start stimulation (Fig. 1)

Ovarian stimulation can be started at any time (“random start stimulation”) [4–7]. According to a study by Kuang et al. [17], pregnancy rates after stimulation start in the luteal phase are similarly high to those after a stimulation start in the early follicular phase and the malformation rate is unaffected [18]. According to studies to date, stimulation with stimulation starting in the luteal phase takes 1–2 days longer, and a slightly higher gonadotrophin dose is required per day than for a stimulation start in the early follicular phase [6, 7].

**Fig. 1:**
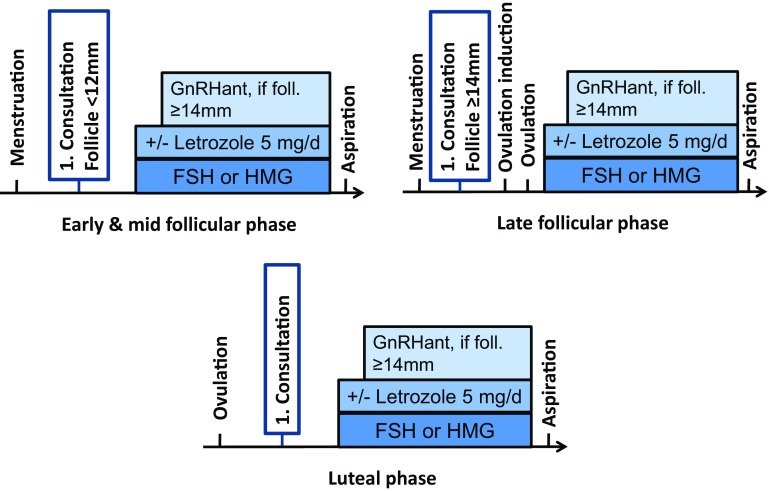
Ovarian stimulation in the early, mid, late follicular, and luteal phases ([5], modified)

Depending on the cycle phase, stimulation can be carried out as follows (Fig. 1):

- Stimulation start in the early to mid follicular phase: the conventional antagonist protocol with HMG or FSH, addition of the antagonist when control follicle ≥ 14 mm and GnRH-agonist triggering with triptorelin 0.2 mg s.c. when 3 follicles ≥ 17 mm. Stimulation dose is approx. 50 IU higher than for an intended fresh transfer.
- Stimulation starts in the late follicular phase with a leading follicle ≥ ca. 14 mm: ovulation induction with follicular size of ca. 14 mm with triptorelin 0.2 mg s.c. followed by luteal phase stimulation immediately after ovulation.
- Stimulation start in the luteal phase: the conventional antagonist protocol with hMG or FSH and GnRH-agonist triggering with triptorelin 0.2 mg s.c. Stimulation dose is slightly higher than after stimulation start in the early follicular phase. Start of antagonists as soon as the newly developed leading follicle is ≥ around 14 mm.

#### Double stimulation

In double stimulation [8], stimulation with a classical antagonist protocol as well as ovulation induction with a GnRH-agonist is initially performed. It can be assumed that the first stimulation can also be started in any cycle phase (random start stimulation). Small follicles are not aspirated. A second stimulation is started around 5 days later. To exclude premature ovulation, GnRH antagonists are administered as soon as the leading follicle exceeds 14 mm. The time required for double stimulation is ca. 30 days.

#### Reducing the estradiol concentration in estrogen-sensitive tumours

To reduce the increasing estrogen concentrations during ovarian stimulation, the addition of aromatase inhibitors, e.g., letrozole 5 mg (2.5 mg each morning and evening from the first day of stimulation) is recommended [19]. The number of mature oocytes obtained and their fertilization capacity is not reduced by the addition of letrozole [5]. The previous studies have not shown increased malformation rates in children after low dose stimulation with letrozole [20].

#### Combination of ovarian stimulation with the removal of ovarian tissue

**Fig. 2:**
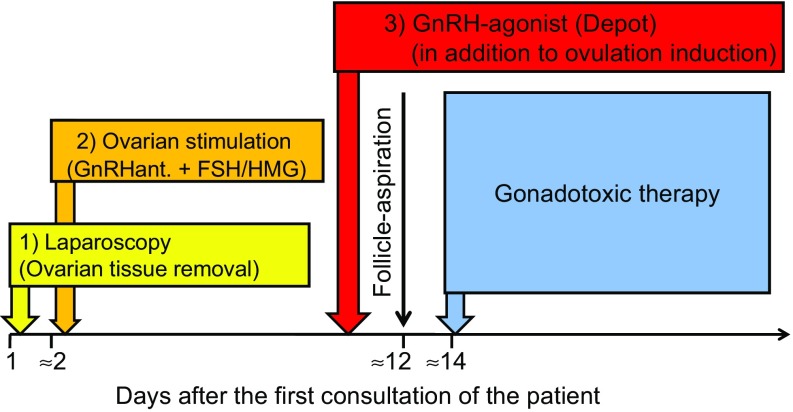
Combination of the three main techniques to preserve fertility before gonadotoxic therapy

Ovarian stimulation can be combined with cryopreservation of ovarian tissue to increase the success rate after highly gonadotoxic treatment (Fig. 2) [9, 21]. 50% of an ovary is removed laparoscopically and ovarian stimulation is started 1–2 days later. According to the studies carried out so far, there is no increased complication risk and the number of oocytes obtained is not significantly reduced after removal of ovarian tissue. The time required for the combination of both treatments is about 2.5 weeks.

## Cryopreservation and transplantation of ovarian tissue

### Background

If there is a sufficiently high ovarian reserve, part of the ovarian tissue can be cryopreserved for later transplantation. Since the cryopreserved tissue volume is not very large and some of the follicles degenerate during cryopreservation and transplantation, the transplants are only active for a few years. Therefore, the transplant does not serve to restore long-term ovarian function, which would replace hormone replacement therapy, but only to achieve a pregnancy. Transplantation for the induction of puberty [22, 23] as well as postponement of the menopause [24] have been reported; however, since transplantations for these indications are of limited use endocrinologically [25], the cryopreservation of ovarian tissue should only be performed to establish a fertility reserve.

In principle, the higher the ovarian reserve (i.e., the follicle density in cryopreserved and transplanted ovarian tissue), the greater the chances of pregnancy; therefore, cryopreservation of ovarian tissue is ideal for younger women or even children. The upper age limit is often stated as 35 years, although this limit can be around 38 years for women with a high ovarian reserve.

### Efficacy

The data for assessing effectiveness are still limited. However, since data from previously published case series show similar success rates, the probability of birth per transplant can already be roughly estimated.

In a case series from Denmark with 41 women, 53 transplantations were performed [26]. A total of 42 transplants were performed in 32 women because of a wish to conceive. 31% of transplanted women gave birth to at least one child.

In a case series from the *Ferti*PROTEKT network, 95 transplants were performed in 74 women [27]. A sub-analysis of the 40 women who received their first transplant for POI included 11 pregnancies and 9 births. This corresponds with birth rate of 23% per transplant.

According to data from Denmark, some pregnancies are generated after repeated transplantations; therefore, a higher success rate per woman is expected in the future. Since transplantation techniques are being further optimized, a similar success rate to ovarian stimulation and cryopreservation of oocytes could possibly achieved.

A case report has been published that reported a birth after the cryopreservation of ovarian tissue at the age of 13 years (premenarchal) and a transplant at the age of 27 [28]. This case report shows that transplantation can lead to pregnancies even after prepubertal cryopreservation of ovarian tissue. This is supported by two case reports, which have shown an induction of puberty by the transplantation of prepubertal cryopreserved ovarian tissue [22, 23].

### Risks

The removal and transplantation of ovarian tissue requires a laparoscopy, the transplant another laparoscopy, possibly a laparotomy. When removing ovarian tissue, there is an increased risk of infection and bleeding depending on the oncological disease (e.g., in leukaemia patients). According to the *Ferti*PROTEKT register, one complication per 500 laparoscopies which necessitates surgical revision is expected for the removal of ovarian tissue [29]. The surgical risks for the transplantation of ovarian tissue are not higher than for any other laparoscopy.

Based on knowledge of the ovarian metastasis potential of oncological diseases and based on systematic studies of cryopreserved ovarian tissue, a preliminary, still incomplete risk category for ovarian metastases has been developed for individual oncological diseases (Table 2). In high-risk cases, cryopreservation of ovarian tissue should be considered as experimental and the patient should be informed that the tissue might not be used or can only be used after further establishment of the techniques mentioned below.

**Table 2:** Risks of ovarian metastasis ([56], modified)

Low risk	Medium risk	High risk
Breast cancer stage I–II and infiltrating ductal subtype Squamous cell carcinoma of the cervix Hodgkin’s lymphoma Osteogenic carcinoma Wilms tumour Non-genital rhabdomyosarcoma	Breast cancer stage IV and infiltrating lobular subtype Colon cancer Adeno carcinoma of the cervix Non-Hodgkin’s lymphoma Ewing sarcoma	Leukaemia Neuroblastoma Burkitt lymphoma Ovarian carcinoma

### Practical approach

#### Removal of ovarian tissue

Before the removal of ovarian tissue, the ovarian reserve should be determined to avoid cryopreservation in women with a low ovarian reserve. Ovarian tissue should not be taken from the side of the corpus luteum, as the quality of the ovarian tissue may be restricted.

Approximately 50% of the ovary is removed from the antimesenteric side using scissors without electrical coagulation. In the *Ferti*PROTEKT network, 50% of the ovarian tissue was removed in 97% of women [10]. The removal of an entire ovary is only recommended for prepubertal girls due to the small size of the ovaries. The wound surface is rinsed to identify the small, mostly subcortical sources of bleeding and to selectively coagulate them. A closure of the wound surface is not necessary. A small sample from the removed ovarian tissue is placed in formalin and handed to the pathologist to exclude metastases.

#### Transportation of tissue

The removed ovarian tissue should be transferred immediately after removal into a sterile transport medium (e.g., Custodiol, Dr. Franz Köhler Chemie, Bensheim, Germany) and transported to the processing site at 4–8 °C. A transport time of up to 22 ± 2 h is possible with constant cooling at 4–8 °C [30, 31], which can be ensured by the special cooling packs and suitable transport containers provided by the respective cryobanks.

#### Preparation and freezing of ovarian tissue

A clean room laboratory with a contamination-free environment and a sterile class II lamina air flow should be available for preparation, where sterile and cooled dissection can be performed. The ovarian medulla should be carefully dissected from the ovarian cortex using a precision scalpel and anatomical forceps and a thin residual stroma should be left, so that an optimal starting point for the neovascularization of the transplants is available later. Depending on the size and quality, rectangular pieces of cortex, ca. 8 × 4 × 1 mm, are cut from the prepared cortex, cooled in a suitable medium for cryopreservation, equilibrated, and then finally transferred to individual cryogenic vials filled with cryomedium. In a computer-controlled slow-freezing process, the samples are cooled according to a modified program by Gosden et al. [32], so that they can be stored indefinitely in liquid nitrogen (at – 196 °C).

#### Transplantation of ovarian tissue

The number of tissue fragments to be transplanted is determined using the ovarian reserve, and if possible the follicular density after histological determination, and the age of the patient at the time of removal of the tissue. The amount of ovarian tissue to be transplanted usually corresponds to approximately 15–25% of an entire ovary.

**Fig. 3:**
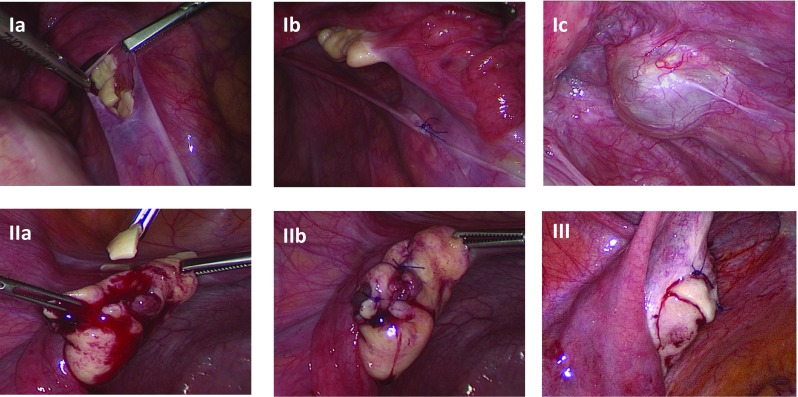
Transplantation of ovarian tissue I: subperitoneally in the ovarian fossa (Ic: 12 months later), II: into the ovary, and III: onto the ovary (University women’s hospital, Bern, Switzerland)

The ovarian tissue is mainly transplanted orthotopically, i.e., into the pelvic wall lateral to the ovaries, into or onto the ovary (Fig. 3). A transplant onto the ovary best imitates the physiological anatomy, but requires the greatest laparoscopic-microsurgical expertise and the duration of surgery (1–2 h) is the longest. A transplant into the pelvic wall is the least physiological localization, but surgically the easiest to perform and the operation only takes about ½–1 h. Which localization leads to the highest chances of pregnancy is still unclear. How much ovarian tissue should be transplanted is also still open to discussion. In the transplantations that led to a pregnancy in the *Ferti*PROTEKT network [27], approximately 15–20% of the amount of an entire ovary was transplanted. During the transplantation, the patency of the tubes should be checked and, if necessary, a hysteroscopy should also be considered.

#### Transplantation into the pelvic peritoneum

A 0.5–1 cm incision is made in the parietal peritoneum lateral to the ovary. A subperitoneal pocket is bluntly dissected and the pieces of tissue are placed, so that the cortex surfaces face the pelvis (Fig. 3, Ia). The pieces of tissue should lie side by side. Peritoneal closure is usually performed with single interrupted sutures (for example, with PDS 6-0) (Fig. 3, Ib), and with fibrin glue if necessary.

#### Transplantation into the ovary

The tissue is usually placed into the larger ovary and thus usually into the organ from which the ovarian tissue did not originate before gonadotoxic therapy. The ovary is incised (Fig. 3, IIa), ideally resulting in a subcortical pocket. The tissue pieces are transplanted into the pocket, so that the cortex surfaces face the ovarian surface if possible. The closure of the ovary is usually performed with sutures (Fig. 3, IIb).

#### Transplantation onto the ovary

A transplant onto the ovary surface requires the tissue pieces to be of a sufficient size, so that they can be fixed. The ovary is, for example, incised on the surface using scissors, which opens a wound gap that is bluntly dilated. The tissue pieces can be fixed onto this with single interrupted sutures (e.g., with PDS 5-0) (Fig. 3, III). Fixation with fibrin glue and coverage with an Interceed^®^ mesh have also been reported [33]. A video demonstrating transplantation of ovarian tissue into the peritoneal wall and onto the ovary is available at the Website of ESHRE (Special interest group “Fertility preservation”), http://www.eshre.eu.

#### Follow-up after transplantation

The first signs of ovarian activity are seen after about 3 months. If the tubes are open and no other relevant sterility factor is present, a spontaneous pregnancy may be attempted. Cycle monitoring is often performed, ovulation is induced with hCG, and the timing of sexual intercourse is optimized. The many spontaneous pregnancies [27] confirm that spontaneous conception can initially be attempted. If a pregnancy does not occur or a different relevant sterility factor is present, then IVF, possibly combined with ICSI, can be carried out. Gonadotropin stimulation for the generation of several follicles can be attempted, but this often does not lead to a multifollicular reaction due to the low ovarian reserve and is also associated with higher costs. Alternatively, a modified “Natural Cycle”-IVF can be performed [34].

According to the transplants in the *Ferti*PROTEKT network, approximately 2/3 of the transplants are still active after 1 year [27].

Since it is currently unclear which transplant location is ideal and how much tissue should be transplanted, the transplantation of ovarian tissue should only be carried out within the scope of clinical trials if possible. *Ferti*PROTEKT is conducting an open international multicentre study to determine the ideal transplant site (https://clinicaltrials.gov/, NCT02780791).

## GnRH agonists (GnRHa)

### Background

The effect of GnRHa is based on the hypothesis that the resulting pituitary downregulation and “inactivation” of the ovarian activity would lead to a reduced sensitivity to cytotoxic effects. However, activation of the primordial to secondary follicles is gonadotropin-independent, so a protective influence cannot be plausibly explained this way [35]. Thus, the mode of action of GnRHa is currently unclear.

### Efficacy

Most meta-analyses since 2011 showed a significantly lower rate of POI occurrence after chemotherapy-accompanying GnRHa administration. The risk can be reduced by about half, admittedly in a heterogeneous data situation [35–39]. A significant influence on the likelihood of a later pregnancy has not been proven so far. Demeestere et al. [40] published the first study on the long-term effect of GnRHa and were unable to demonstrate a benefit with a median follow-up of 5.3 years after chemotherapy. There is currently still a need for clarification and the need for complementary prospective randomized studies on the use of GnRHa for this indication.

### Risks

In principle, GnRHa can lead to climacteric symptoms such as hot flushes, etc. However, during chemotherapy, the ovarian function is suppressed anyway, and such symptoms are possible a few days earlier at the most because of the previously administered GnRHa.

The irreversible loss of bone density, which is possible if GnRHa is administered for > 6 months, is normally irrelevant here, as chemotherapy generally does not exceed this time. Regardless of this, it should be noted that GnRHa may theoretically affect the efficacy of chemotherapy on estrogen-sensitive tumours. This negative effect on chemotherapy has not been confirmed, however, but was rather disproved.

### Practical approach

After the initial GnRHa application, a gonadotropin flare up is noted. However, whether this flare up is relevant is currently unclear, as the primordial follicles are not gonadotropin sensitive.

GnRHa are usually administered s.c. or i.m. as monthly or 3-monthly depots.

A depot-GnRHa should be repeatedly injected, so that the downregulation lasts about 1–2 weeks beyond the end of the last chemotherapy cycle.

## Ovarian transposition

### Background

The aims of ovarian transposition are the preservation of hormonal ovarian function and the possibility of pregnancy, even after oncological treatment. The effects of radiotherapy on ovarian function are considerable. A dose of 2 Gy to the ovaries (LD50) reduces the follicular density by half [41]. The dose for induction of a premature ovarian insufficiency in a 30-year-old woman is specified as 16 Gy [42]. Ovarian transposition should be considered when targeted radiotherapy is performed in the pelvic area. In addition to the benefits and risks, alternatives such as cryopreservation should be discussed. A combination of different fertility preservation techniques is also possible.

Ovarian transposition is not appropriate in patients who undergo total body irradiation. Pelvic radiotherapy is often performed in Hodgkin’s and non-Hodgkin’s lymphoma, rectal cancer, Ewing’s sarcoma of the pelvis, and cervical cancer.

The question of whether ovarian transposition should be performed prior to radiotherapy can only be decided individually. In addition to the expected gonadal toxicity, the possible wish for a unilateral transposition plays a role to allow spontaneous conception via the remaining ovary.

### Efficacy

The results of ovarian transposition before radiotherapy depend on various factors and are, therefore, difficult to quantify. In a meta-analysis of 32 publications with a total of 1189 patients, the success rate in the sense of preserved ovarian function is stated as 80.8% (17–95%) [43]. Publication bias is to be assumed, since many cases or studies with a poor success rate may not be published [44].

Furthermore, the success rate of ovarian transposition is also determined by the surgical technique. Different techniques such as cranial, lateral, medial, and anterior transposition have been described [45]. Due to the inhomogeneity of the cases and the absence of prospective randomized studies, it is not possible to make a reliable statement on the comparison of the different techniques, whereby cranial transposition is the safest technique for reducing the dose of radiation.

However, the importance of the new position of the ovaries has been proven. The distance from the radiation field is decisive, since 10% of the radiation dose is still active at a distance of 10 cm from the radiation field [46]. Radiotherapy to the entire pelvis, despite transposition, is significantly more likely to lead to ovarian failure than localized after loading (35 versus 6%) [47]. In this respect, close collaboration between the surgeon and radiologist is necessary before the planned transposition.

The positioning height of the ovary is also a relevant prognostic factor. In a multivariate analysis, the positioning height with an odds ratio of 11.7 was the most relevant prognostic factor for the ovarian function. The ovary should lie at least 2 cm above the iliac crest [48]. In addition, a safety margin of approximately 2 cm must be included, because the position of the ovaries can change postoperatively [49].

Overall, ovarian transposition is assumed to be highly effective in the sense of preserving ovarian function. However, pregnancies are rare [50]. On one hand, patients may no longer wish to have children after completing oncological treatment [51]. On the other hand, radiotherapy to the uterus significantly reduces pregnancy chances. The radiation-induced alteration of the endometrium also influences the success rate of cryopreservation of the ovaries before radiotherapy.

### Risks

The surgical risks of ovarian transposition are low. In most cases, the procedure is possible via laparoscopy. If laparotomy is performed because of another indication, ovarian transposition can be carried out simultaneously without a substantial increase in the complication rate. Ovarian tissue can also be removed during this procedure for cryopreservation. Ovarian cysts sometimes develop postoperatively which are a sign of disturbed ovarian function [43]. In most cases, however, these cysts do not require treatment.

### Practical approach

The infundibulum is prepared cranially until tension-free fixation of the ovary in the desired position is possible. Coagulation and/or torsion of the ovarian vessels must be avoided. Blood flow can be well controlled by inspection of the tube, whereas this is not possible with the ovary. The ovary is placed in the desired position, e.g., with single interrupted sutures and marked with metal clips for radiotherapy planning. To avoid intestinal obstruction, the vascular pedicle should also be fixed to the abdominal wall.

### In vitro maturation und experimental techniques

There are numerous other techniques in addition to those mentioned. Most of these techniques are still experimental (Table 3). Only in vitro maturation (IVM) is also used in humans.

**Table 3:** Experimental fertility preservation techniques

Technique	Detail	Effectiveness in animal model	Effectiveness in human system
Xenotransplantation [31]	Transplantation of ovarian tissue into immunodeficient animals for to generate oocytes, especially in diseases with a high risk of tumour cell contamination of ovarian tissue	Development of offspring in mouse/rat model	Oocytes were obtained after transplantation into SCID mice. So far, no generation of embryos
In vitro growth (IvG) [57]	Cultivation of ovary tissue or isolated follicles to generate oocytes, especially in diseases with a high risk of ovarian tumour cell contamination	Development of offspring in mouse model	Development of embryos from primates in the mouse. Development of human germinal vesicles in the mouse
“Artificial ovary” [58, 59]	Isolation of preantral follicles from ovarian tissue, fixation in a matrix and orthotopic transplantation of the matrix Especially in diseases with a high risk of ovarian tumour cell contamination	Development of follicles in the mouse	No application in the human system as yet

For IVM, the chances of success with a fresh transfer are relatively high in some centres. However, after additional oocyte cryopreservation, which is necessary for use as a fertility preservation measure, the success rates are low. Cao et al. [52] reported that only 13% of in vitro-matured, cryopreserved, and fertilized oocytes developed into embryos compared to 33% without prior cryopreservation. Roesner et al. [53] reported only 2 births after 32 thawing cycles in 61 cases where in vitro maturated oocytes had been cryopreserved. IVM is, therefore, not very effective and should only be performed in women with a very high antral follicle count (AFC) and only if the 2 weeks required for gonadotropin stimulation mentioned above are not available.

Alternatively, IVM can be used to aspirate the oocytes from the follicles in ovarian tissue prior to its cryopreservation. Huang et al. [54], reported on 4 women, aged 21–38 years. A partial oophorectomy was performed in 3 women and a total oophorectomy in one. A total of 11 oocytes were aspirated from the removed tissue of the 4 women, of which 8 oocytes could be maturated and vitrified. Uzelac et al. [55] reported on a 23-year-old from whom 10 immature oocytes were collected after an unilateral oophorectomy; four of which were matured. The cryopreservation, however, only took place after fertilization of the oocytes. 2 embryos were later transferred and a pregnancy developed that resulted in a birth. These and other case reports show that only very few oocytes can be collected from removed ovarian tissue. Furthermore, if the above-mentioned very low blastocyst development rates [52] and birth rates [53] are considered, it is evident that this method is not very effective.

Various techniques are being developed to enable the use of ovarian tissue in patients with haematological cancers with the purpose of avoiding the transmission of tumour cells. However, these techniques, which are shown in Table 3, are still purely experimental.
